# Supplementary material for: Morpho-agronomic, biochemical and molecular analysis of genetic diversity in the Mesoamerican common bean panel
Source: PLoS One. 2021 Apr 22;16(4):e0249858. doi: 10.1371/journal.pone.0249858 (PMC8062103; doi:10.1371/journal.pone.0249858)
Supplement: S1 Table — (DOCX) [file pone.0249858.s001.docx]

**Table 1**. List of accessions constituting the Mesoamerican Panel of Bean Diversity (MPBD).

| Accessions | Genetic Material | Developing institution^1/^ | Seed Color |
| --- | --- | --- | --- |
| Diamante_Negro | Cultivars | EMBRAPA | black |
| Aporé | Cultivars | EMBRAPA | carioca |
| BRS_Ametista | Cultivars | EMBRAPA | carioca |
| BRS_Campeiro | Cultivars | EMBRAPA | black |
| BRS_Esplendor | Cultivars | EMBRAPA | black |
| BRS_Esteio | Cultivars | EMBRAPA | black |
| BRS_Estilo | Cultivars | EMBRAPA | carioca |
| BRS_Expedito | Cultivars | EMBRAPA | black |
| BRS_FC104 | Cultivars | EMBRAPA | carioca |
| BRS_FC402 | Cultivars | EMBRAPA | carioca |
| BRS_FP403 | Cultivars | EMBRAPA | black |
| BRS_Grafite | Cultivars | EMBRAPA | black |
| BRS_Horizonte | Cultivars | EMBRAPA | carioca |
| BRS_Notável | Cultivars | EMBRAPA | carioca |
| BRS_Pitanga | Cultivars | EMBRAPA | purple |
| BRS_Pontal | Cultivars | EMBRAPA | carioca |
| BRS_Requinte | Cultivars | EMBRAPA | carioca |
| BRS_Supremo | Cultivars | EMBRAPA | black |
| BRS_Valente | Cultivars | EMBRAPA | black |
| BRSMG_Madrepérola | Cultivars | EMBRAPA | carioca |
| BRSMG_Pioneiro | Cultivars | EMBRAPA | carioca |
| BRSMG_Talismã | Cultivars | EMBRAPA | carioca |
| Bambuí | Cultivars | EMBRAPA | beige |
| Guapo_Brilhante | Cultivars | EMBRAPA | black |
| Macanudo | Cultivars | EMBRAPA | black |
| Minuano | Cultivars | EMBRAPA | black |
| Ouro_Negro | Cultivars | UFV/EPAMIG | black |
| Pérola | Cultivars | EMBRAPA | carioca |
| Rudá | Cultivars | EMBRAPA | carioca |
| Safira | Cultivars | EMBRAPA | red |
| FT_120 | Cultivars | FT Sementes | black |
| FT_NOBRE | Cultivars | FT Sementes | black |
| FT_41 | Cultivars | FT Sementes | black |
| FT_65 | Cultivars | FT Sementes | carioca |
| FT_Soberano | Cultivars | FT Sementes | black |
| IAC_Akitã | Cultivars | IAC | carioca |
| IAC_Alvorada | Cultivars | IAC | carioca |
| IAC_Carioca | Cultivars | IAC | carioca |
| IAC_Diplomata | Cultivars | IAC | black |
| IAC_Formoso | Cultivars | IAC | carioca |
| IAC_Imperador | Cultivars | IAC | carioca |
| IAC_Milênio | Cultivars | IAC | carioca |
| IAC_Sintonia | Cultivars | IAC | carioca |
| IAC_Una | Cultivars | IAC | black |
| IAC-Carioca_Aruã | Cultivars | IAC | carioca |
| IAC-Carioca_Pyatã | Cultivars | IAC | carioca |
| IAC-Carioca_Tybatã | Cultivars | IAC | carioca |
| IAC-Maravilha | Cultivars | IAC | black |
| Moruna | Cultivars | IAC | black |
| IAPAR_16 | Cultivars | IAPAR | cream |
| RAI_214 | Cultivars | IAPAR | black |
| IAPAR_57 | Cultivars | IAPAR | carioca |
| IAPAR_65 | Cultivars | IAPAR | black |
| IAPAR_14 | Cultivars | IAPAR | carioca |
| IAPAR_20 | Cultivars | IAPAR | black |
| IAPAR_31 | Cultivars | IAPAR | carioca |
| IAPAR_72 | Cultivars | IAPAR | carioca |
| IAPAR_80 | Cultivars | IAPAR | carioca |
| IAPAR_81 | Cultivars | IAPAR | carioca |
| IPR_139_Juriti_Claro | Cultivars | IAPAR | carioca |
| IPR_Andorinha | Cultivars | IAPAR | carioca |
| IPR_Bem-te-vi | Cultivars | IAPAR | carioca |
| IPR_Campos_Gerais | Cultivars | IAPAR | carioca |
| IPR_Celeiro | Cultivars | IAPAR | carioca |
| IPR_Chopim | Cultivars | IAPAR | black |
| IPR_Colibri | Cultivars | IAPAR | carioca |
| IPR_Corujinha | Cultivars | IAPAR | carioca |
| IPR_Curió | Cultivars | IAPAR | carioca |
| IPR_Eldorado | Cultivars | IAPAR | carioca |
| IPR_Gralha | Cultivars | IAPAR | black |
| IPR_Graúna | Cultivars | IAPAR | black |
| IPR_Juriti | Cultivars | IAPAR | carioca |
| IPR_Maracanã | Cultivars | IAPAR | carioca |
| IPR_Inhambu | Cultivars | IAPAR | black |
| IPR_Quero-quero | Cultivars | IAPAR | carioca |
| IPR_Sabiá | Cultivars | IAPAR | carioca |
| IPR_Saracura | Cultivars | IAPAR | carioca |
| IPR_Siriri | Cultivars | IAPAR | carioca |
| IPR_Tangará | Cultivars | IAPAR | carioca |
| FEB200 | Breeding Lines | CIAT | black |
| IPR_Tuiuiú | Cultivars | IAPAR | black |
| IPR_Uirapuru | Cultivars | IAPAR | black |
| IPR_Urutau | Cultivars | IAPAR | black |
| Rio_Iguaçu | Cultivars | IAPAR | black |
| Rio_Negro | Cultivars | IAPAR | black |
| Rio_Pardo | Cultivars | IAPAR | cream |
| Rio_Piquiri | Cultivars | IAPAR | brown |
| Rio_Tibagi | Cultivars | IAPAR | black |
| Rio_Red | Cultivars | IAPAR | red |
| Rio_Doce | Cultivars | IAPAR | carioca |
| HF_465.63.1 | Cultivars | IPA | cream |
| IPA_1 | Cultivars | IPA | cream |
| IPA_10 | Cultivars | IPA | black |
| IPA_6 | Cultivars | IPA | brown |
| IPA_7 | Cultivars | IPA | cream |
| IPA_74-19 | Cultivars | IPA | cream |
| IPA_9 | Cultivars | IPA | brown |
| Princesa | Cultivars | IPA | carioca |
| TAA_Bola_Cheia | Cultivars | TAA | carioca |
| TAA_Dama | Cultivars | TAA | carioca |
| TAA_Gol | Cultivars | TAA | carioca |
| Awauna | Cultivars | UEM | black |
| Flor_Diniz | Cultivars | UEM | carioca |
| Rico23 | Cultivarsr | UFV | black |
| Campeão | Cultivars | Agristar | carioca |
| Agronorte_09 | Cultivars | Agronorte | carioca |
| ICA_Pijão | Cultivars | ICA | black |
| ICA_Quetzal | Cultivars | ICA | black |
| ICA_Tui | Cultivars | ICA | black |
| Iratin | Landraces | NI | black |
| Emgopa_Ouro | Cultivars | Incaper | cream |
| A775 | Breeding Lines | CIAT | cream |
| A779 | Breeding Lines | CIAT | cream |
| AETE2 | Cultivarsr | IAC | cream |
| ARC1 | Breeding Lines | CIAT | black |
| ARC2 | Breeding Lines | CIAT | black |
| BAT1215 | Breeding Lines | CIAT | red |
| BAT40 | Breeding Lines | CIAT | black |
| BAT41 | Breeding Lines | CIAT | red |
| BAT451 | Breeding Lines | CIAT | black |
| BAT58 | Breeding Lines | CIAT | black |
| BAT76 | Breeding Lines | CIAT | black |
| BAT1192 | Breeding Lines | CIAT | red |
| BAT477 | Breeding Lines | CIAT | brown |
| Black_Hawk | Cultivars | MSU | black |
| BZ16987 | Breeding Lines | NI | cream |
| Carioca1070 | Breeding Lines | CENA/USP | carioca |
| DOR191 | Breeding Lines | CIAT | red |
| DOR351 | Breeding Lines | CIAT | purple |
| DOR365 | Breeding Lines | CIAT | carioca |
| DOR445 | Breeding Lines | CIAT | black |
| DOR446 | Breeding Lines | CIAT | black |
| DOR483 | Breeding Lines | CIAT | red |
| DOR500 | Breeding Lines | CIAT | black |
| DOR364 | Breeding Lines | CIAT | cream |
| EMP250 | Breeding Lines | CIAT | carioca |
| ESAL583 | Breeding Lines | ESALQ | carioca |
| FEB149 | Breeding Lines | CIAT | cream |
| FEB151 | Breeding Lines | CIAT | cream |
| FEB156 | Breeding Lines | CIAT | cream |
| FEB159 | Breeding Lines | CIAT | cream |
| G1261 | Landraces | CIAT | red |
| G14866 | Landraces | CIAT | black |
| G17666 | Landraces | CIAT | cream |
| G18141 | Cultivarsr | CIAT | gust |
| G2358 | Landraces | CIAT | white |
| G2676 | Cultivarsr | CIAT | black |
| G3593 | Landraces | CIAT | red |
| G4002 | Landraces | CIAT | carioca/black |
| G4825 | Landraces | CIAT | carioca |
| G5285 | Cultivarsr | CIAT | red |
| G5902 | Landraces | CIAT | black |
| FEB178 | Breeding Lines | CIAT | carioca |
| MD732 | Breeding Lines | IAPAR | cream |
| Michigan | Cultivarsr | MSU | white |
| MUS49 | Breeding Lines | CIAT | red |
| MUS80 | Breeding Lines | CIAT | red |
| NAB87 | Breeding Lines | CIAT | black |
| NEP171 | Breeding Lines | IICA | black |
| PORRILLO_70 | Cultivarsr | CIAT | black |
| Porrillo_Sintético | Cultivarsr | CIAT | black |
| RIZ57 | Breeding Lines | CIAT | carioca |
| RJR21 | Breeding Lines | NI | white |
| Rosinha_G1 | Landraces | IAC | cream |
| Roxinho_Ivaí | Landraces | NI | black |
| Purple_de_Mato_Grosso | Landraces | NI | purple |
| Purple_de_Minas | Landraces | NI | purple |
| XAN206 | Breeding Lines | CIAT | black |
| XAN236 | Breeding Lines | CIAT | black |
| LP03 | Breeding Lines | IAPAR | carioca |
| LP04 | Breeding Lines | IAPAR | carioca |
| LP05 | Breeding Lines | IAPAR | carioca |
| LP06 | Breeding Lines | IAPAR | carioca |
| LP07 | Breeding Lines | IAPAR | carioca |
| LP08 | Breeding Lines | IAPAR | carioca |
| LP09 | Breeding Lines | IAPAR | carioca |
| LP10 | Breeding Lines | IAPAR | carioca |
| LP11 | Breeding Lines | IAPAR | carioca |
| LP12 | Breeding Lines | IAPAR | carioca |
| LP13 | Breeding Lines | IAPAR | black |
| LP14 | Breeding Lines | IAPAR | black |
| LP15 | Breeding Lines | IAPAR | black |
| LP16 | Breeding Lines | IAPAR | black |
| LP17 | Breeding Lines | IAPAR | black |
| LP18 | Breeding Lines | IAPAR | carioca |
| LP19 | Breeding Lines | IAPAR | carioca |
| LP20 | Breeding Lines | IAPAR | black |
| LP21 | Breeding Lines | IAPAR | carioca |
| LP22 | Breeding Lines | IAPAR | carioca |
| LP23 | Breeding Lines | IAPAR | carioca |
| LP24 | Breeding Lines | IAPAR | carioca |
| LP25 | Breeding Lines | IAPAR | carioca |
| LP26 | Breeding Lines | IAPAR | carioca |
| LP27 | Breeding Lines | IAPAR | carioca |
| LP28 | Breeding Lines | IAPAR | carioca |
| LP29 | Breeding Lines | IAPAR | black |
| LP30 | Breeding Lines | IAPAR | black |
| LP31 | Breeding Lines | IAPAR | black |
| LP32 | Breeding Lines | IAPAR | black |
| LP33 | Breeding Lines | IAPAR | black |
| LP34 | Breeding Lines | IAPAR | carioca |
| LP35 | Breeding Lines | IAPAR | black |
| LP36 | Breeding Lines | IAPAR | carioca |
| LP37 | Breeding Lines | IAPAR | carioca |
| LP38 | Breeding Lines | IAPAR | carioca |
| LP39 | Breeding Lines | IAPAR | carioca |
| LP40 | Breeding Lines | IAPAR | black |
| LP41 | Breeding Lines | IAPAR | carioca |
| LP42 | Breeding Lines | IAPAR | carioca |
| LP43 | Breeding Lines | IAPAR | black |
| LP44 | Breeding Lines | IAPAR | black |
| LP45 | Breeding Lines | IAPAR | black |
| LP46 | Breeding Lines | IAPAR | black |
| LP47 | Breeding Lines | IAPAR | carioca |
| LP48 | Breeding Lines | IAPAR | carioca |

^1^CIAT = International Center for Tropical Agriculture, EMBRAPA = Brazilian Agricultural Research Corporation (Empresa Brasileira de Pesquisa Agropecuária), IAC = Agronomic Institute of Campinas (Instituto Agronômico de Campinas), IAPAR = Rural Development Institute of Paraná – IAPAR – EMATER (Instituto de desenvolvimento Rural do Paraná).
