# Supplementary material for: Morpho-agronomic, biochemical and molecular analysis of genetic diversity in the Mesoamerican common bean panel
Source: PLoS One. 2021 Apr 22;16(4):e0249858. doi: 10.1371/journal.pone.0249858 (PMC8062103; doi:10.1371/journal.pone.0249858)
Supplement: Attachment — Submitted filename: Reviewer Recommendation and Comments.pdf [file pone.0249858.s002.pdf]

Specific considerations:

The manuscript needs to be formatted according to PLOS ONE's style requirements, mainly the Heading, quotes from the figures (Fig 1 and not Figure 1), and line spacing. The standards are listed in the link:

https://journals.plos.org/plosone/s/file?id=wjVg/PLOSOne_formatting_sample_main_body.pdf

If the authors used the abbreviation "i.e.," and "e.g.," for the texts between "()", the work would be a better fit.

Probably, using the word "Brazilian" in the title is not correct since 20% of the accessions are of foreign origin (CIAT).

ABSTRACT

The summary is well written; however, a main conclusion is missing.

L35 – The word "heritability" is duplicated.

L43 - Delete "AFLP".

INTRODUCTION

The introduction is well written and covers the main topic of the study. However, there was a lack as to the importance of using the three types of analysis (Morpho-agronomic, biochemical, and molecular).

L57-L60 – The citation ([6]) does not support the first three arguments of the sentence (1° one of the world's largest producers and consumers of beans; 2° Mesoamerican cultivars are more popular; 3° carioca and black commercial types are preferred, accounting for 85 and 11% of Brazil's bean production). Provide references.

L67 – Change "variation and potential" by "diversity".

L67 – Change "characteristics" by "traits".

L70 – Change “such accessions must be fully characterized and evaluated” by “the candidate accessions must be fully evaluated and characterized, both morphologically and molecularly.”

L71 – Change “exploitation” by “exploration”.

L71-74 – Maybe would look better with a sentence like that (just a suggestion): “The precise exploration of genetic diversity by the breeding programs is necessary for the development of new commercial cultivars adapted to the most diverse regions of the country. Usually, higher levels of diversity in the set used for breeding is related to a greater chance of identifying higher agronomic traits [12, 13].”

L74 – Change “[12] [13]” by “[12,13]”.

L75- Change “The common bean exhibits wide variation in a variety of traits” by “The common bean exhibits wide agronomic traits variation”.

L82- Change “various” by “several”.

L83 – Change “[12] [16]” by “[12,16]”.

MATERIALS AND METHODS

For a better understanding of the study, the authors should provide more details about the panel. Why were the panel accesses selected? What percentage of Cariocas, black, and colored beans? How many are commercial cultivars and how many are breeding lines?

Another important point, based on what information is it possible to assure that the selected accessions are Mesoamerican?

The description of the “Morpho-agronomic characterization” section could be improved. Were all evaluations performed after the harvest? How many plants from the useful plot were evaluated?

The description of the “Molecular characterization” section is very extensive, if possible, they could only mention the changes made in relation to the study cited “Vos et al. [25]”.

The “Data analysis” and “Biochemical characterization” section is really good.

L94 – add “from different breeding programs and countries.”

L97 – add the sowing month of the experiment.

L105 – Change “([18])” by “[18]”.

L153 – Change “Amplified fragment length polymorphism (AFLP)” by “AFLP”, the abbreviation has already been defined in the summary.

RESULTS

The results are clear and well presented.

The values of Ac and h^2 (YLD) need to be revised, figure 1 shows one value and the text describes another. Another point, the abbreviation “FT (total phenolic contents)” is like “TP” in figures 1, 2 and 3.

L222 – Change “The Ac values were high (≥ 0.70) for most traits but relatively low (0.52) for YLD (Figure 1).” by “The Ac values were high (≥ 0.70) for all traits, except for YLD (0.52)”.

L223 – Change “Meanwhile, h^2 was high for DPPH, FT,224 FLA, W100, and NN, low for YLD (0.27), and intermediate (0.59–0.68) for all other traits” by “Meanwhile, h^2 was high (0.98 – 0.90) for DPPH, FT, FLA, W100, and NN, intermediate (0.59–0.79) for all other traits, except for YLD (0.15).”

L250 – “of the selected accessions” Which accessions were selected? Or does it refer to the panel?

L268 and L272 – The IFP “cm” unit is missing.

L309 and 335 – Add more details to the legend (e.g., the traits used for analysis, grouping method, and the colors of the figure).

DISCUSSION

L345 – The panel was not “established”. Change by “evaluated”.

L355 – Change “for DPPH, FLA, FT, W100, and NN, high (□ 0.70) for SP, PL, IFP, and STL, and low for YLD.” by “and high (□ 0.70) for the other traits, except YLD, which was low.”

361 – Change “The low Ac value obtained for YLD may hinder the selection of accessions. The variable was highly influenced by the environment and was reflected in the heritability” by “The low Ac value obtained for YLD is a consequence of the high influence of the environment for the trait, making it difficult to select the best accessions.”

362 – add “also” after “et al. [40]”.

L386 – Change “PerseguinI’ by “Perseguini”

L394, 407 and 409 – “the word "topologies" sounds strange.

L410 – Change “for future genetic breeding programs” by “to be exploited by genetic breeding programs.”

REFERENCES

L414 – Wrong reference, perhaps the correct one is: “Yeken MZ, Akpolat H, Karaköy T, Çiftçi V 2018. Assessment of Mineral Content Variations for Biofortification of the Bean Seed. International Journal of Agricultural and Wildlife Sciences. 4(2):261-9.”

L465 - ???

L466 - ???

L478 - Uppercase names.

L483- ???

L491 – incomplete

FIGURES

The quality of Figure 4 is poor, it would be better to increase the space between the labels.

SUPPORTING INFORMATION

Change “Description” by “Details”

“gene group”??

Change “access” by “accessions”.

Table title needs to be improved.

Translate “Obtentor”.

Change “Commercial Group” by “Seed Color”.

Provide the meaning of each abbreviation in the “Obtentor” column in the Footnotes.

Add a column with the average of the morphological and biochemical traits of each accessory.

Correct cultivar names: IPR_Nhambu, IPR_NIriri, RoNInha_G1, IAC_NIntonia, and Porrillo_NIntético.
